# An outbreak of schistosomiasis in a primary school in Omusati region, Namibia, March, 2016

**DOI:** 10.11604/pamj.supp.2018.30.1.15263

**Published:** 2018-05-16

**Authors:** Kofi Mensah Nyarko, Uzenia Ndatelela Mupakeleni, Francine Ananias, Emmy-Else Ndevaetela, Joseph Asamoah Frimpong

**Affiliations:** 1 Namibia Field Epidemiology and Laboratory Training Program, Windhoek, Namibia; 2 Ministry of Health and Social Services, Namibia; 3 African Field Epidemiology Network, Accra, Ghana vc

**Keywords:** Outbreak investigation, viral, exanthema, Nigeria

## Abstract

Schistosomiasis is endemic in some parts of northern Namibia and there is a control program in the country with the use of mass drug administration to control and prevent the disease. On the 1st March, 2016, there was a report of bloody urine among primary school pupils in a school in Omusati region, Namibia. A team of health professionals was dispatched to investigate. This case study describes steps in conducting a schistosomiasis outbreak investigation and how to determine the risk factors. This describes how to calculate both the basic and analytical measures of association with 95% confidence intervals. This case study provides a step-by-step approach and can be used as a tool to teach the fundamental principles of outbreak investigation and response and how to measure the appropriate measures of association. This case study is targeted at intermediate- and advanced-level residents of the Field Epidemiology and Laboratory Training Program and other epidemiology trainees.

## How to use this Study

**General instructions:** ideally, 1 to 2 instructors facilitate the case study for 15 students in a classroom or conference room. The instructor should direct participants to read a paragraph out loud, going around the room to give each participant a chance to read. When the participant reads a question, the instructor directs all participants to perform calculations, construct graphs, or engage in discussions. The instructor may split the class to play different roles or take different sides in answering a question. As a result, participants learn from each other, not just from the instructors. There are also specific instructor’s notes that are included with each question in the instructor’s version of this case study.

**Audience:** residents in the 9-month intermediate and the 2-year advanced Field Epidemiology Training Programs (FETP), Masters of Public Health Training Programs, and others who are interested in this topic.

**Prerequisites:** before using this case study, participants should have received lectures or other instruction in outbreak investigation, epidemiological study designs and measures of association.

**Materials needed:** laptop with Microsoft Excel, Epi-info or graph paper, flipchart or white board with markers and calculators

**Level of training and associated public health activity:** Intermediate and Advanced – Outbreak investigation

**Time required:** approximately 3 hours

**Language:** English

## Competing interest

The authors declare no competing interest.
